# Trajectories and determinants of emergency department use among nursing home residents: a time series analysis (2012–2019)

**DOI:** 10.1186/s12877-022-03078-4

**Published:** 2022-05-12

**Authors:** Gianmarco Giacomini, Ettore Minutiello, Gianfranco Politano, Marco Dalmasso, Beatrice Albanesi, Sara Campagna, Maria Michela Gianino

**Affiliations:** 1 Department of Public Health Sciences and Pediatrics, Università di Torino, Via Santena 5 bis, 10126 Torino, Italy; 2 Department of Control and Computer Engineering, Politecnico di Torino, Torino, Italy; 3 Unit of Epidemiology- Local Health Unit TO3, Via Sabaudia 164, Turin, Grugliasco Italy

**Keywords:** Emergency departments, Nursing home, Determinants

## Abstract

**Background:**

Emergency department (ED) use among nursing home (NH) residents is an internationally-shared issue that is understudied in Italy. The long term care in Italy is part of the health system. This study aimed to assess trajectories of ED use among NH residents and determinants between demographic, health supply, clinical/functional factors.

**Methods:**

A pooled, cross-sectional, time series analysis was performed in an Italian region in 2012/2019. The analysis measured the trend of ED user percentages associated with chronic conditions identified at NH admission. A GLM multivariate model was used to evaluate determinants of ED use. The variables collected were sex, age, assistance intensity, destination after discharge from NH, chronic conditions at NH admission, need for daily life assistance, degree of mobility, cognitive impairments, behavioural disturbances and were taken from two databases of the official Italian National Information System (FAR and C2 registries) that were combined to create a unique and anonymous code for each patient.

**Results:**

A total of 37,311 residents were enrolled; 55.75% (20,800 residents) had at least one ED visit. The majority of the residents had cardiovascular (25.99%) or mental diseases (24.37%). In all pathologies, the percentage of ED users decreased and the decrease accelerated over time. These results were confirmed in the fixed effects regression model (coefficient for linear term (b = − 3.6177, *p* = 0, 95% CI = [− 5.124, − 2.1114]); coefficient for quadratic term = − 0.7691, *p* = 0.0046, 95% CI = [− 1.2953, − 0.2429]). Analysis showed an increased odds of ED visits involving males (OR = 1.27, 95% CI 1.24;1.30) and patients affected by urogenital diseases (OR = 1.16, 95% CI [1.031–1.314]). The lowest odds of ED visits were observed among subjects aged > 90 years (OR = 0.64, 95% CI [0.60–0.67]), who required assistance for their daily life activities (OR = 0.86; 95% CI = [0.82, 0.91]), or with serious cognitive disturbances (OR = 0.86; 95% CI = [0.84, 0.89]), immobile (OR = 0.93; 95% CI = [0.89, 0.96]), or without behavioural disturbances (OR = 0.92; 95% CI = [0.90, 0.94]).

**Conclusions:**

The percentage of ED users has decreased, through support from the Italian disciplinary long-term care system. The demographic, clinical/functional variables associated with ED visits in this study will be helpful to develop targeted and tailored interventions to avoid unnecessary ED use.

## Background

The World Health Organization defines long-term care (LTC) as “all activities undertaken by others to ensure that people with, or at risk of, a significant ongoing loss of capacity can maintain a level of functional ability consistent with their basic rights, fundamental freedoms, and human dignity” [1]. Long-term care can be provided in different places: at home, in the community, and in a facility such as a nursing home.

The Italian LTC public system is organized around two institutional pillars: home and residential care services. Residential care offers care to multiple categories of patients who have the following types of clinical conditions: nonself-sufficient patients; people with mental disorders; minors with psychiatric and neurodevelopmental disorders; people with pathological addictions (drug and alcohol); and people with disabilities.

Residential care for non self-sufficient patients is provided by nursing homes (NHs) [2], all the other clinical conditions (i.e. people with mental disorders; minors with psychiatric and neurodevelopmental disorders; people with pathological addictions; and people with disabilities) are treated by other types of facilities.

Residential care in NHs is managed by local health units (ASLs, Aziende Sanitarie Locali) that, after the request of the general practitioner and following an overall assessment of the physical, psychological and social conditions by a multidimensional assessment unit, authorizes admission to a NH.

Patients of any age can be admitted to the NH, although the majority are ≥65 years old. NH offers multiple levels of care intensity and guarantees a length of stay generally until death, even if the residential care can be stopped by hospital admission or transfer to another type of facility or to home.

At the moment of enrolment into a NH, a formal individual care plan is created by a multi-professional team.

The aim of admission to the NH is to guarantee a comprehensive, coordinated approach across different actors to improve their clinical and functional status, minimize their symptoms, improve their quality of life and avoid unnecessary hospital admission and emergency department (ED) visits [2].

ED visits from nursing home residents are an issue that is shared internationally [3, 4], and although EDs are necessary for acute management, many transfers can be avoided through better clinical management of patients in residential facilities [5, 6]. Moreover, it is preferable to reduce unnecessary visits by long-term patients, who often have a high mortality rate due to the complexity of their clinical cases [7]. NH residents were also found to be more prone to complications during transfer and visit to the emergency room, such as a decline in cognitive and physical condition or contracting hospital-acquired infections [8]. A review has identified a number of potentially modifiable patient and organizational factors that should reduce the need for burdensome transfer to the ED and improve the quality of care for this population of frail individuals [9]..

From this perspective, it could be useful to evaluate whether patients enrolled in NHs have reduced ED visits over time and to identify the determinants of ED use. As far as we know, the ED visits of NH residents are understudied in Italy. Therefore, to bridge this gap, this study aimed to analyse the trends and trajectories in the last decade of the percentage of NH residents using EDs (EDusers) in the Italian health care system, investigating determinants for admission to the EDs among demographic, health supply and clinical/functional variables.

## Methods

A pooled, cross-sectional, time series analysis was performed over an eight-year period (2012/2019) among nursing home residents in Piedmont, Italy.

### Context

As described above, residential care in NHs is managed by local health units that authorize admission to a NH after the request of a general practitioner and an assessment of the physical, psychological and social conditions of the patient by a multidimensional assessment unit. The multidimensional assessment unit assigns a score to the patient through the use of the following rating scales: functional Barthel rating scale to autonomy in activities of daily living (ADL); Barthel mobility assessment scale to Degree of mobility; S.P.M.S.Q.-Short Portable Mental Status rating scale to Cognitive disorders and A.DI.CO, Area of Disorders Behavioral to Behavioral disorders. Based on a national conversion table score/intensity of care, the multidimensional assessment unit defines which of the formal levels of intensity of care is necessary.

At the moment of enrolment in the NH, a formal individual care plan is created with the purpose of mapping needed care and guaranteeing adequate care in relation to the patient’s needs and the provision of all the necessary devices and aids. Formal care plan is formulated and provided by a team of general practitioners or own doctors, specialists, nurses, physiotherapists/speech therapists and social-health workers. If NHs have not own entire professional team dedicated, the ASLs provide healthcare workers needed. A clinical record is opened at the time a patient is admitted to the NH and closed at the time of the patient’s death or after being transferred to the home or hospital or to other types of residential facilities.

### Data sources

All residents enrolled in NH between 2012/2019 were recruited.

All ED visits that occurred among NH residents were accounted for during the period studied.

The following variables were collected. Demographic characteristics of residents: sex; age (< 66, 66–80, 81–90, > 90); Health supply variable: assistance intensity (low and medium/low, high and medium/high); destination after discharge from the NH (at home, admitted to hospital, other type of residential facilities, dead in NH, no discharge); and residents’ clinical and functional variables: chronic conditions identified when the older person was first admitted to NH (categorized according to the International Classification of Diseases 9th revision: cardiovascular diseases; digestive system diseases; endocrine and metabolic diseases; haematological diseases; infectious diseases; neoplasms; mental disorders; musculoskeletal diseases; neurologic disorders; perinatal and congenital disorders that are congenital pathologies or those arising in the perinatal phase or in very early childhood; respiratory diseases; trauma; urogenital diseases; and other diseases); need for assistance in daily life (independent, needs some help, dependent); degree of mobility (independent, walks with help of one person, immobile); cognitive impairments (absent, moderate, serious); behavioural disturbances (present, absent).

All data were obtained from two information sources: the Nursing home residents’ information system (FAR registry) database (which is the official Italian National Information System created for monitoring residential care and is administered by local health units that manage residential care in NH) and the Italian National Information System for ED use database (C2 registry).

All data come from the Health Information System of the Piedmont Region, designed and administered to comply with the regional, national and European regulations regarding the protection of personal data and used in support of the evaluation and monitoring activities of the Piedmont Region.

Data from these databases were merged using the universal patient ID number, an anonymous, unique code centrally assigned to each patient, before data storage. Moreover, the data treatment phases have been exclusively performed by operators of the Regional epidemiology network (of which the Unit of Epidemiology, Regional Health Service, Local Health Unit TO3 is a node), in charge of the processing of pseudonymised data to support regional evaluation, as stated in the Regional regulation (DGR 10 January 2012, n. 3–3259 – ‘Disciplinary of the modalities of access to the regional health information assets’) and authorized to access the data by Resolution n. 976 of 20 November 2014’Governance of the Regional Health Information System - Role and operating methods of the Epidemiology network” by the Health Department - Health Information Systems Sector. The cooperation between the units involved in this study is regulated by a formal agreement. Therefore, ethics committee approval was unnecessary.

### Statistical analysis

The entire analysis was performed with the R framework [10], and the significance level was set at *p* < 0.05. We resorted to a linear regression estimate of ED user percentages per year for the patients involved in NHs to compute a time-trend analysis.

The analysis measured the trend of ED user percentages associated with chronic conditions identified when the older person was first admitted to the NH. Each trend was modelled as a regression, and both linear and quadratic terms were taken into account. We took advantage of Beck and Katz Robust Covariance Matrix Estimators to correct for Robust Standard Errors for Panel Models, and eventually, to assess robustness, we resorted to the t-Wald test of estimated coefficients. For each significant trend, we extracted the beta coefficient as a model of the overall slope of the trend.

Furthermore, to determine the possible associations between the selected independent variables (demographic, health supply, and clinical/functional) and the chance of at least one ED visit, we resorted to a pooled, cross-sectional, time series analysis with fixed-effect estimation. We computed an overall fixed-effect model and tested it against a random-effect model by the mean of the Hausman test [11, 12]. In both models, we took advantage of using robust estimators for the standard errors to assess the significance of each predictor. By resorting to the Arellano method [13], we corrected the standard estimation of predictors with a heteroskedasticity-consistent estimation of the covariance matrix of the coefficient. By adding dummy variables to the model for each year of the study period but the first, we checked for any possible exogenous time trends involving both the independent and dependent factors (i.e., time-fixed effects), thus assessing time-invariant heterogeneity for the model.

We computed the odds of a list of determinants possibly associated with ED use. The considered determinants were age, sex, chronic conditions identified when the older person was first admitted to the NH, assistance intensity, need for assistance in daily life, mobility, cognitive impairment, and behavioural disturbances. We computed the odds taking advantage of a GLM multivariate model with a Poisson bias function to properly deal with the count data produced by counting multiple ED visits per person. The GLM function did consider all the determinants thus far, producing a model that was able to infer the odds ratio of recurrent accesses among determinants. Due to the high amount of variables and strata, thus possible permutations, we have computed odds adjusted and we reported non- combined effects of determinants. Statistical significance was set at *p* < 0.05, and 95% confidence intervals (CIs) were also reported.

## Results

### Emergency department users percentage

A total of 37,311 patients were enrolled in NHs during the study period (2012–2019).

A total of 73.29% of the residents were ≥ 81 years old and only 2.98% were < 66 years old (ages 55–65). 72.06% were female, and 64.90% required high to medium/high assistance intensity. The most commonly reported chronic conditions identified when the older person was first admitted to the NH were mental disorders (25.99%), cardiovascular diseases (24.37%), and neurological disorders (15.67%). Fifty-three percent of residents experienced high to medium/high needs for their daily life, 60.17% were immobile, and 43.35 and 48.61% experienced serious cognitive impairment or the presence of behavioural disturbances, respectively. Sixty-six percent died in NH (Table 1). The median length of stay was 489 days.

**Table 1:** Baseline characteristics of patients receiving residential care (*N* = 37,311) who visited the Emergency Department (ED)-2012/2019

Variables	N.	%
**Age**		
< 66	1112	2,98
66–80	8852	23,72
81–90	18,291	49,02
> 90	9056	24,27
**Sex**		
Female	26,886	72,06
Male	10,425	27,94
**Chronic conditions identified at NH admission**		
Other diseases	372	1,00
Cardiovascular diseases	9092	24,37
Digestive system diseases	314	0,84
Endocrine and metabolic diseases	1778	4,77
Haematological disorder	117	0,31
Infectious diseases	126	0,34
Mental disorders	9697	25,99
Missing	5913	15,85
Musculoskeletal diseases	910	2,44
Neoplasms	757	2,03
Neurological disorders	5847	15,67
Perinatal and congenital disorders	169	0,45
Respiratory diseases	874	2,34
Trauma and injury	1029	2,76
Urogenital diseases	316	0,85
**Assistance intensity**		
Low and medium/low	13,098	35,10
High and medium/high	24,213	64,90
**Need for assistance in daily life**		
Independent	1542	4,13
Needs some help	10,850	29,08
Dependent	19,621	52,59
Missing	5298	14,20
**Mobility**		
Independent	4013	10,76
Walks with help of one person	5550	14,87
Immobile	22,450	60,17
Missing	5298	14,20
**Cognitive impairments**		
Absent	6322	16,94
Moderate	9515	25,50
Serious	16,176	43,35
Missing	5298	14,20
**Behavioural disturbances**		
Present	18,138	48,61
Absent	13,875	37,19
Missing	5298	14,20
**Destination after discharge from NH**		
Home	567	1,52
Admitted to Hospital	849	2,28
Other type of residential facilities	1078	2,89
Dead in NH	24,452	65,54
No discharge	10,365	27,78

**Fig. 1:**
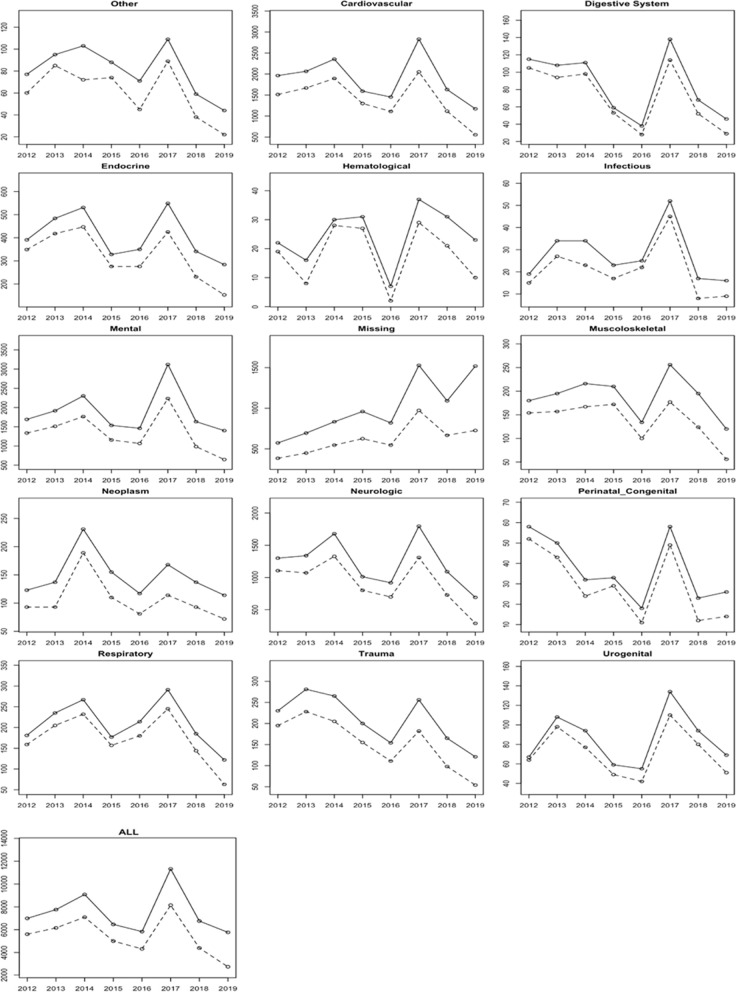
Number of patients enrolled in NH who reported at least one ED visit by pathology at NH admission and from 2012 to 2019. Solid line: Number of patients enrolled in NH over time; dashed lines – number of patients who reported ED visits over time. NH –Nursing Home care; ED – Emergency Department

Among our cohort of 37,311 residents, 20,800 (55.75%) reported at least one ED visit over an eight-year period. Figure 1 shows that the variability in ED user percentages across pathologies was high: the percentage never dropped below 30% for any pathology, and 3 pathologies: digestive, neoplasms and urogenital, had an ED user percentage higher than 60% during the analysed period.

### Annual trends in the percentage of ED users

**Fig. 2:**
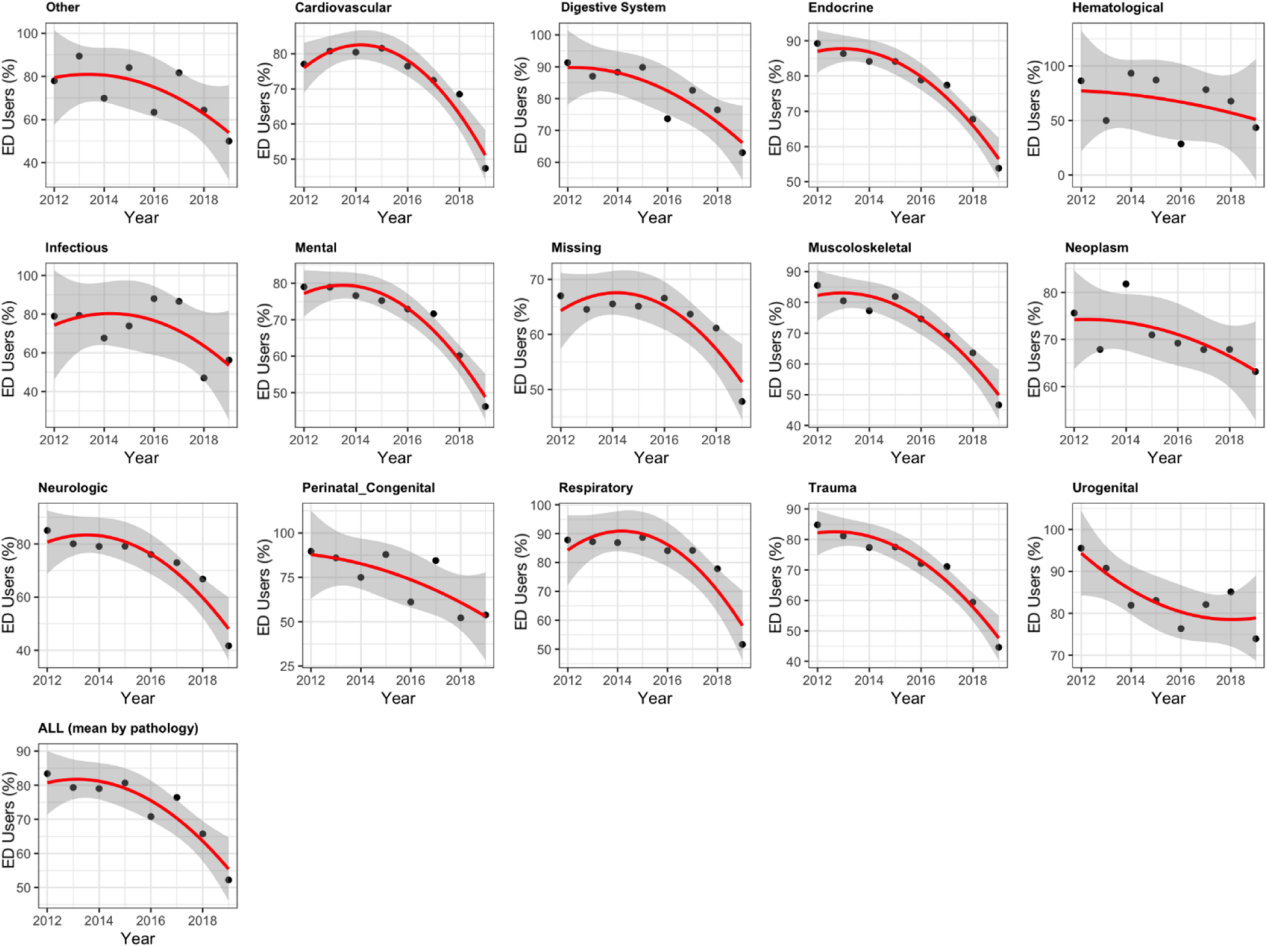
Fixed-effects regression analysis. ED user percentages (%), by pathologies at NH admission and from 2012 to 2019. NH –Nursing Home care; ED – Emergency Department

In all pathologies, the percentage of ED users decreased, and the decrease accelerated over time. These results were confirmed in the fixed effects regression model. The linear trend (b = − 3.6177, *p* = 0, 95% CI = [− 5.124, − 2.1114]) was significant and trend was significantly “curved” (coefficient for quadratic terms = − 0.7691, *p* = 0.0046, 95% CI = [− 1.2953, − 0.2429]). This meant that the average annual percentage decreased over the 8-year study period and that this decrease accelerated over time (Fig. 2) because when the coefficient for the linear term is negative and the coefficient for a quadratic term is less negative and significant, it means that the time trend is convex and that the value at the end of the study period is significantly lower than that at the beginning. Most pathologies had a convex trend with a high decrease, such as neurologic disorders, respiratory diseases, cardiovascular diseases, endocrine and metabolic diseases, trauma, mental diseases and musculoskeletal diseases, whereas digestive diseases and perinatal diseases had a convex trend with a slight decrease.

Infectious, neoplasms and emathological diseases experienced a reverse trend but no acceleration in the decrease in the percentage of ED users; only urogenital diseases showed a concave trend.

### Determinants of emergency department use

Regression analysis showed significant results.

Among the demographic variables, the analysis showed an increased risk of ED visits involving males (OR = 1.27, 95% CI 1.24;1.30), whereas ORs decreased with increasing age, with the lowest risk of ED visits observed among subjects aged > 90 years (OR = 0.64, 95% CI [0.60–0.67]) (Table 1).

Patients affected by urogenital diseases showed the highest risk for ED visits (OR = 1.16, 95% CI [1.03–1.31]) (Table 2). Patients who were dependent on assistance for their daily life, had serious cognitive impairments, were immobile, or did not have behavioural disturbances had a lower likelihood for an ED visit (Table 1).

**Table 2:** Results of the regression analysis

Variables	Adjusted OR^**a**^	95% CI
**Age**		
< 66	1	
66–80	0.95	[0.90–1.00]
81–90	0.82	[0.78–0.86]
> 90	0.64	[0.60–0.67]
**Sex**		
Female	1	
Male	1.27	[1.24–1.30]
**Chronic conditions identified at NH admission**		
Other diseases	1	
Cardiovascular diseases	0.85	[0.77–0.93]
Digestive system diseases	1.08	[0.96–1.22]
Endocrine and metabolic diseases	0.91	[0.83–1.01]
Haematological disorder	0.85	[0.71–1.02]
Infectious diseases	0.80	[0.67–0.96]
Mental disorders	0.75	[0.68–0.82]
Missing	0.80	[0.72–0.90]
Musculoskeletal diseases	0.84	[0.76–0.94]
Neoplasms	0.82	[0.74–0.92]
Neurological disorders	0.82	[0.74–0.90]
Perinatal and congenital disorders	0.82	[0.70–0.96]
Respiratory diseases	1.06	[0.95–1.17]
Trauma and injury	0.81	[0.73–0.90]
Urogenital diseases	1.16	[1.03–1.31]
**Assistance intensity**		
Low and medium/low	1	
High and medium/high	0.99	[0.96–1.00]
**Need for assistance in daily life**		
Independent	1	
Needs some help	0.98	[0.93–1.02]
Dependent	0.86	[0.82–0.91]
**Mobility**		
Independent	1	
Walks with help of one person	1.01	[0.98–1.05]
Immobile	0.93	[0.89–0.96]
**Cognitive impairments**		
Absent	1	
Moderate	0.95	[0.92–0.97]
Serious	0.86	[0.84–0.89]
**Behavioural disturbances**		
Present	1	
Absent	0.92	[0.90–0.94]

*NH* nursing home care, *OR* odds ratio, *CI* 95% confidence interval

^a^OR of one or more ED visits, computed by GLM analysis

## Discussion

This study aimed to analyse the trajectories in the last decade of the percentage of ED users among NH residents in the Italian health care system, investigating determinants for admission to the EDs among demographic, health supply and clinical/functional variables.

The analysis showed a decline in the percentage of ED users over the past 8 years. A decrease was reported for all chronic conditions at the NH admission, even though it occurred in a heterogeneous manner. It is not easy to explain the reason for this significant decline, also because there have been no specific policy actions that would have resulted in substantial changes to care delivery over time. However some explanations may account for this finding. This result may have been facilitated by strengthening of the type of care model adopted in the Italian health care system, which is characterized by some fundamental elements. First, the multidimensional evaluation of the person; second, the drafting of a care plan by a multiprofessional and multidisciplinary team. This model consents to provide integration of care, clinical review, support, and management of the acutely or chronically unwell resident in NH. This suggestion is in keeping with findings from previous studies showing that Care models that enhance the nursing home’s capacity to provide on-site evaluation and management of acute changes through early recognition, monitoring, and staff have been shown to decrease the number of ED visits among nursing home residents without increased mortality rates [14, 15].

This result may have been influenced by the type of health care system within which our study took place. The National Health Service (NHS), by Laws: March 11th 1988, n. 67 – art. 20 and Decree of the president of the council of ministers (DPCM) on January 12, 2017 [2], guarantees, through public resources, the opportunity to stay in nursing homes to people who are not self-sufficient and who are unable to care for themselves at their home. The suggestion that our results may be affected by the type of health care system is in agreement with a previous study reporting that for older people (≥ 75 years), the availability of beds in nursing homes was inversely associated with ED visit rates [16]. This suggestion is reinforced considering that, in our study, most of the residents were over 80 years old.

According to the results, some residents’ demographic characteristics and functional variables reduce odds to ED visits.

The results showed that a nonfrequent ED user may be > 90 or be female.

Although it is already known that males are more predisposed to ED visits [8], a review concluded that the influence of age is not very clear in the literature due to the heterogeneity of outcomes and methods of most of the studies, mostly because studies used different age categories or assessed age as a continuous variable [8]. However, other literature confirms our results. A review reported that ED visits seemed to decline with increasing age, especially > 80–85 years [17], and similarly, a study identified NH residents younger than 75 years as a group at greatest risk for ED visits [18].

Additionally, according to our study, residents with severe impairment have lower odds of ED use. This finding is not surprising and consistent with those of previous studies. Compared with residents with mild cognitive impairment (CI), an American study in 2011 [19] showed that the adjusted odds ratio of ED visits decreased as the severity of CI increased and that residents with severe CI had up to a 40% lower odds of visiting the ED [20]. Another study reported that advanced CI appears to be protective against the odds of ED visits [21]. The literature offers different suggestions to explain this result. On the one hand, this suggests that residents with mild severity CI have a higher risk of visiting the ED, probably because those individuals were not yet been formally diagnosed with a cognitive impairment. In the early stages of CI, patients may exhibit symptoms that create the uncertainty regarding how to treat them, thereby leading to transfer of the resident to the ED [20]. Alternatively, the literature suggests that the result of a decreasing ED visit trend for those who have severe CI may be explained by the good recognition of the negative consequences for this vulnerable group of ED transfer that would accelerate their functional declines [21]. To these suggestions, it can be added that it is more likely that patients suffering severe cognitive impairment are treated with a more palliative or with a comfort care approach, making them less likely to visit the ED [18, 19].

Not surprisingly, residents dependent on ADL appeared to have reduced odds of ED use. This result is consistent with previous studies showing that residents who had moderate ADL dependence had a greater odds of making ED visits [18, 22]. It is realistic to assume that residents with greater ADL need are the subject of greater attention from the team of health workers in order to avoid future ED use for falls, skin disorders, and dehydration occurring with greater frequency among older adults who do not have fully met ADL [23].

Another important and expected result was that the absence of problem behaviours reduced the odds of ED use. Indeed, having behavioural problems, such as apathy, agitation or aggression, is known to be associated with a diminished quality of life for NH residents, as well as worse patient–physician/nurse care relationships. In addition, behavioural problems are often treated with antipsychotics, which may have negative side effects [24, 25]. In essence, either for their presence or their treatment, behavioural problems increase the complexity of care and the professional uncertainty surrounding the proper treatment [22], increasing the need for ED use.

To our knowledge, this is the first study to examine ED user trends among NH residents over almost a decade in a large Italian region. The findings of the present study have multiple policy implications. First, the declining trends of ED users suggest NH managers foster an assistance model based on a holistic approach that is able to offer continuity of care and ad hoc treatment for every patient’s need, either physical, psychological or social. Indeed, in Italy, this holistic approach was supported by the DPCM 12/01/2017, [4] which provides for a multidimensional assessment that considers clinical, psychological and sociofamily dimensions and explores the various factors that can configure the condition of fragility through various qualitative and quantitative measuring instruments. The multidimensional assessment allows the redaction of the individualized care plan. This plan in Italy is one of the minimum organizational requirements for a NH, and it is a fundamental document that summarizes the conditions in which the person finds himself and defines a personalized approach developed to favour a condition of life, health and dignified well-being. In addition, this plan is provided by a team that guarantees medical care delivered by the general practitioner or own doctors and specialist care provided by the healthcare workers of ASL. This access to medical care supports avoidance of ED visits.

Second, the declining trends of ED users seem to confirm the effectiveness of the LTC model, it should be implemented by policy-makers, keeping in mind the importance of reducing transfers for elderly or fragile patients due to their negative consequences in terms of mortality and adverse clinical effects [9] A positive push in this direction is given in Italy thanks to the incoming Piano Nazionale di Ripresa e Resilienza (PNRR), which provides funding for fundamental matters such as digital health and telemedicine that also comprehends eConsults with specialists and other professionals [26]. In this way, it will become easier to monitor and treat patients more frequently in the same structure and daily setting, providing them with more stability and quality of care. This means that eConsults improve access to specialist advice, even containing costs, and provide a way to reduce ED use that requires family assistance or specialized services because of patients’ frailty [27, 28]. Some studies have demonstrated the feasibility of telemedicine in NHs to reduce ED utilization. These studies report a reduction of ED usage by 18–46%, without increasing GP visits or mortality [3].

The results and implications of this study must be considered in light of the study’s limitations. The main limitations are those of the databases used and are common to all administrative database studies. First, there are problems related to the quality of the NH data, especially with regard to the possible lack of accuracy and completeness of information across individuals and institutions. Even taking this weakness into account, these databases are the best available sources, suitable for wide epidemiological studies on the prevalence and incidence of major diagnoses or diseases and for monitoring population trends in the utilization of services. Another limitation of this study is that the NH residents were drawn from one country. Nevertheless, we have gathered information on a large cohort of patients over a long observation period across settings. Due to the study being performed in a large northern Italian region that has a health care system and population health profiles comparable to those of other European countries [29], our results may be helpful and useful for providing a comparison to other future analyses of ED visits from other countries.

## Conclusion

In Italy, the percentage of ED users among NH residents has decreased over time, through support the Italian disciplinary long-term system. Keeping in mind the importance of reducing unnecessary ED visits of NH residents because of the well-known negative consequences in terms of mortality and adverse effects, this result can be further improved through the support from NH managers and policy-makers. The demographic and clinical/functional variables associated with ED visits in this study will be helpful to develop targeted and tailored interventions to avoid unnecessary ED use.

## Data Availability

Datasets used in the current study are not publicly available due to embedded legal policies. Data are managed and were provided by the Unit of Epidemiology, Regional Health Service, Local Health Unit TO3, Piedmont Region, Italy. Any access to datasets is granted to allowable bodies upon written agreement between involved institutions. Requests can be addressed to the attention of the director prof. Giuseppe Costa giuseppe.costa@epi.piemonte.it.

## Abbreviations

ED: Emergency Department
NH: Nursing Home
LTC: Long-term care
CI: Cognitive impairment
DPCM: Decreto del Presidente del Consiglio dei Ministri
PNRR: Piano Nazionale di Ripresa e Resilienza
